# I can’t stop using cocaine and my son is going to be taken away from me

**DOI:** 10.1192/j.eurpsy.2022.2121

**Published:** 2022-09-01

**Authors:** A. Osca Oliver, V. Ros Font, M.V. López Rodrigo, M. Palomo Monge, M.F. Tascón Guerra, M. Pérez Fominaya

**Affiliations:** 1 Hospital Nuestra Señora del Prado, Psiquiatría, Talavera de la Reina, Spain; 2 Hospital Nuestra Señora del Prado, Psiquiatria, Talavera de la Reina, Spain

**Keywords:** cocaine, behaviour, Substance Abuse Detection, Pregnancy

## Abstract

**Introduction:**

The use of psychoactive substances in pregnancy has a similar profile to the general population, in which ethyl alcohol and tobacco are the most widely used drugs, followed, to a much lesser extent, by marijuana and cocaine. Cocaine is a powerful stimulant of the Central Nervous System. Like other smokable cocaines, PBC is highly fat soluble and rapidly crosses the blood-brain barrier, causing maternal-fetal harm when consumed during pregnancy. Being its pathophysiological mechanism the vasoconstriction of uterine and fetal vessels. Obstetric complications related to this toxic mechanism of action include: increased risk of spontaneous abortion, premature detachment of the normal inserted placenta, and intrauterine growth restriction.

**Objectives:**

We present how was the management of a 26-year-old woman, polytoxic, unemployed, living in a “squatting house”, referred from the Gynecology and Obstetrics service to the Addictive Behavior Unit, due to fetal alterations seen in ultrasound follow-ups. Presenting the fetus: delayed intrauterine growth, and bilateral ventriculomegaly with dilation of the left ventricle.

**Methods:**

We report this case to social services and we started doing a weekly poison check. Presenting positive controls for both: cannabis and cocaine.

**Results:**

Due to the physical, psychological and environmental situation of the patient, the withdrawal of custody of her child is being considered.

**Conclusions:**

These types of cases must be treated in a multidisciplinary way, with awareness of the disease and the consequences of continuing to consume must be addressed.

**Disclosure:**

No significant relationships.

